# Sequential Co-immobilization of Enzymes in Metal-Organic Frameworks for Efficient Biocatalytic Conversion of Adsorbed CO_2_ to Formate

**DOI:** 10.3389/fbioe.2019.00394

**Published:** 2019-12-06

**Authors:** Yan Li, Liyin Wen, Tianwei Tan, Yongqin Lv

**Affiliations:** Beijing Key Laboratory of Bioprocess, College of Life Science and Technology, Beijing University of Chemical Technology, Beijing, China

**Keywords:** metal-organic framework, sequential co-immobilization of enzymes, storage of CO_2_, CO_2_ reduction, improved conversion

## Abstract

The main challenges in multienzymatic cascade reactions for CO_2_ reduction are the low CO_2_ solubility in water, the adjustment of substrate channeling, and the regeneration of co-factor. In this study, metal-organic frameworks (MOFs) were prepared as adsorbents for the storage of CO_2_ and at the same time as solid supports for the sequential co-immobilization of multienzymes via a layer-by-layer self-assembly approach. Amine-functionalized MIL-101(Cr) was synthesized for the adsorption of CO_2_. Using amine-MIL-101(Cr) as the core, two HKUST-1 layers were then fabricated for the immobilization of three enzymes chosen for the reduction of CO_2_ to formate. Carbonic anhydrase was encapsulated in the inner HKUST-1 layer and hydrated the released CO_2_ to ${\text{HCO}}_{3}^{-}$. Bicarbonate ions then migrated directly to the outer HKUST-1 shell containing formate dehydrogenase and were converted to formate. Glutamate dehydrogenase on the outer MOF layer achieved the regeneration of co-factor. Compared with free enzymes in solution using the bubbled CO_2_ as substrate, the immobilized enzymes using stored CO_2_ as substrate exhibited 13.1-times higher of formate production due to the enhanced substrate concentration. The sequential immobilization of enzymes also facilitated the channeling of substrate and eventually enabled higher catalytic efficiency with a co-factor-based formate yield of 179.8%. The immobilized enzymes showed good operational stability and reusability with a cofactor cumulative formate yield of 1077.7% after 10 cycles of reusing.

## Introduction

High emissions of greenhouse gas such as CO_2_ into the atmosphere have caused global environmental concern. To obtain a sustainable society, it is highly desirable to explore energy-efficient technologies for the conversion of CO_2_ to useful chemicals or fuels (e.g., methanol). Up to date, considerable efforts have been made to catalyse the hydrogenation of CO_2_ via chemical (Fogeron et al., 2018; Lim et al., 2019), electrochemical (Wang et al., 2016; Coskun et al., 2017; Miller et al., 2019), photochemical (Bachmeier et al., 2014; Tu et al., 2014; Wang D. et al., 2014; Wang and Wang, 2015; Xu et al., 2015; Sokol et al., 2018; Zhang L. et al., 2018), enzymatic conversions (Yang Z.-Y. et al., 2012; Fixen et al., 2016; Shah and Imae, 2017; Cai et al., 2018; Liu et al., 2018), or photocatalyst/biocatalyst integrated systems (Yadav et al., 2014). Compared with other methods, the transformation of CO_2_ to methanol by enzymatic catalysis is preferable due to the significant advantages of high selectivity and specificity, high efficiency, and mild operational conditions (Obert and Dave, 1999; Wang X. et al., 2014; Ji et al., 2015; Kuk et al., 2017; Nabavi Zadeh et al., 2018; Zhang Z. et al., 2018).

The reduction of CO_2_ to methanol by enzymatic cascade reactions mainly involves three enzymes, formate dehydrogenase (FateDH), formaldehyde dehydrogenase (FaldDH), and alcohol dehydrogenase (ADH) (Obert and Dave, 1999; Wang X. et al., 2014; Ji et al., 2015; Kuk et al., 2017; Nabavi Zadeh et al., 2018; Zhang Z. et al., 2018). FateDH converts CO_2_ to formic acid, which is subsequently reduced to formaldehyde catalysed by FaldDH. And formaldehyde is further converted to methanol by ADH at the final step. Although this enzymatic cascade reaction features high specificity, it has a relatively low yield with a methanol conversion of merely 43.8% reported by Dave et al. (Obert and Dave, 1999). The possible rate-limiting step is the first reaction in the sequence catalysed by FateDH since the reaction rate of formic acid oxidation is 30 times faster than CO_2_ reduction (Rusching et al., 1976). One of the conceivable reasons is the low substrate concentration due to the limited solubility of CO_2_ in water. As a result, the increase of CO_2_ substrate concentration in the solution may accelerate the forward conversion of CO_2_ to formic acid. This assumption was well-demonstrated by Zhang et al. who adopted ionic liquids with high CO_2_ solubility to assist the multi-enzymatic conversion of CO_2_ to methanol (Zhang Z. et al., 2018). The yield was increased to approximate 3.5-fold compared to the parallel control experiments.

Metal-organic frameworks (MOFs) belong to the category of organic-inorganic hybrid porous materials built from the coordination between organic linkers and metal ions as nodes (James, 2003; Long and Yaghi, 2009; Tranchemontagne et al., 2009). Compared with conventional porous materials, MOFs possess the advantages of ultrahigh surface area and porosity, uniform and controllable pore sizes, structural diversity, as well as diverse chemistry. The superior properties of MOFs facilitate their wide applications in various research areas. In particular, MOFs are porous materials desired for the adsorption and storage of gases, such as CH_4_, H_2_, and CO_2_ (Li et al., 2009, 2014; Murray et al., 2009; Farha et al., 2010; Liu et al., 2012; Yang S. et al., 2012; Chaemchuen et al., 2013; He et al., 2014; Tian et al., 2017). In this respect, we envisioned that the transformation of CO_2_ to formic acid catalysed by FateDH may also be speeded up if CO_2_ is adsorbed in MOFs and used as substrate. On the other hand, MOFs are also ideal solid supports for the immobilization of enzymes as they can maintain the biological activity of enzymes even under denaturing conditions (Lykourinou et al., 2011; Chen et al., 2012, 2018; Lyu et al., 2014; Gkaniatsou et al., 2017; Lian et al., 2017; Du et al., 2018; Liang et al., 2019). As a result, we intend to develop a MOF platform aiming at achieving the simultaneous storage of CO_2_ and co-immobilization of multienzymes for enhanced cascade reduction of adsorbed CO_2_ to formic acid.

Amine-functionalized MOFs are considered as a promising candidate to enhance CO_2_ capture capacity as the electronegative N atom has a strong affinity to the positive C atom of CO_2_. Tethering amine functionalities in MOFs can be realized by introducing the amine groups on unsaturated metal sites. Chromium(III) terephthalate MIL-101 has a three-dimensional framework consisting of two types of zeotypic mesopores connected by two microporous windows (Férey et al., 2005; Jhung et al., 2007). Except for its distinct merits such as large pore volume, high BET surface area, and excellent stability in water, MIL-101 also contains numerous potential open chromium sites (up to 3.0 mmol/g) (Hwang et al., 2008) with an unoccupied orbital that are expected to anchor amine functionalization via a strong binding interaction with the positive nitrogen atoms. It is also demonstrated that amine-functionalized MIL-101 has high CO_2_ capture capacities (Lin et al., 2013; Yan et al., 2013; Hu et al., 2014; Lin J.-L. et al., 2014; Cabello et al., 2015; Darunte et al., 2016; Huang et al., 2016; Emerson et al., 2018; Zhong et al., 2018; Liu et al., 2019). Thus, in our work, MIL-101(Cr) was fabricated and modified with a series of amines to achieve the efficient storage of CO_2_ substrate.

Three enzymes were chosen for the transformation of CO_2_ to formic acid, carbonic anhydrase (CA), formate dehydrogenase (FateDH), and glutamate dehydrogenase (GDH). The introduction of CA is to accelerate the hydration of CO_2_. Moreover, CO_2_ is a thermodynamically stable molecule with low reactivity, so the conversion of CO_2_ to methanol requires energy which is supplied by co-factor nicotinamide adenine dinucleotide (NADH). GDH was involved into the biocatalysis integrated system to achieve the continuous regeneration of NADH co-factor. To obtain multienzyme systems with enhanced activity, three principles are considered, substrate channeling, kinetics matching, and spatial distribution (Garcia-Galan et al., 2011; Zhang et al., 2015; Walsh and Moore, 2019). The current challenge for the design of multienzyme conjugates remains in the development of efficient strategies realizing the accurate control of enzyme positioning and spatial organization (Fu et al., 2012; Schoffelen and van Hest, 2012; Lin J.-L. et al., 2014). To conquer this limitation, in this work, we adopted a layer-by-layer self-assembly approach to achieve the sequential co-immobilization of multi-enzymes using MOFs in layered structure as the solid scaffold.

**Scheme 1:**
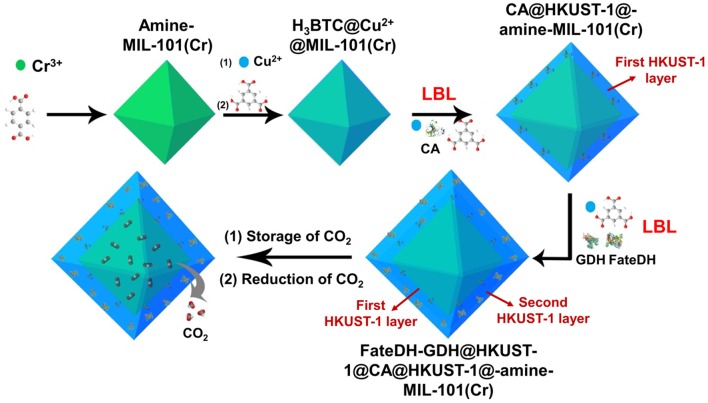
Schematic illustration of the preparation of HKUST-1@amine-MIL-101(Cr)-based multienzymes for the reduction of adsorbed CO_2_.

As illustrated in Scheme 1, amine-functionalized MIL-101(Cr) was first prepared for the adsorption of CO_2_ as substrate. The amine functionalities in MIL-101(Cr) then chelated Cu^2+^ via the formation of a complex followed by further coordinating with 1,3,5-benzenetricarboxylic acid (H_3_BTC). These reactions provided a high density of Cu^2+^ and H_3_BTC on the MOF surface, which then functioned as nucleation sites for the direct formation of HKUST-1 (Hong Kong University of Science and Technology) layers. On the surface of H_3_BTC@Cu^2+^@MIL-101(Cr), the first HKUST-1 layer encapsulated with CA was fabricated using a co-precipitation method via the self-assembly of metal ions, organic linkers, and enzymes. Based on the first HKUST-1 layer, the second HKUST-1 shell immobilizing FateDH and GDH was constructed using the identical approach. In this respect, when CO_2_ was gradually released from MIL-101(Cr), it got access to carbonic anhydrase and was hydrated to bicarbonate ion. The second HKUST-1 layer containing FateDH and GDH directly converted bicarbonate ion to formic acid. The presence of GDH in the second MOF layer achieved the continuous regeneration of NADH co-factor. We found that this sequential co-immobilization route significantly accelerated the cascade biocatalysis reaction rate. The increase of concentration of CO_2_ substrate by storing in MIL-101(Cr) also remarkably boosted the conversion yield.

## Experimental

### Materials and Reagents

Terephthalic acid (99%), trimesic acid (99%), cystamine hydrochloride, and 2,3,4,5,6-pentafluorobenzyl bromide (99%) were purchased from J&K Scientific Ltd. (Beijing, China). Chromium nitrate, copper(II) acetate, and L-glutamic acid were bought from Sinopharm Chemical Reagent Co., Ltd. (Beijing, China). Carbonic anhydrase (CA, bovine red blood cell), formate dehydrogenase (FateDH, lyophilized), and glutamate dehydrogenase (GDH, bovine liver) were provided by Sigma-Aldrich (St. Louis, MO, USA). Nicotinamide adenine quinone dinucleotide (NADH, 98%) was obtained from Aladdin Biotechnology Co., Ltd. (Shanghai, China). CO_2_ (>99%) and ^13^CO_2_ (>99%) were purchased from Beijing Ruyuan Ruquan Technology Co., Ltd. (Beijing, China). All other chemicals were obtained from Beijing Chemical Factory (Beijing, China). Double-distilled water was used in all experiments.

### Instrumentation

The transmission electron microscopy (TEM) images of MOFs were performed using a JEOL 2100F transmission electron microscope (Hitachi, Ltd., Japan). Scanning electron microscopy (SEM) images of MOFs were taken with a JEOL JSM-6700F field emission scanning electron microscope (Hitachi High-Technologies, Tokyo, Japan). Elemental analysis of HKUST-1@amine-MIL-101(Cr) was carried out using an energy dispersive X-ray spectrometer Quantax 200 XF 5010 (Bruker, Germany). Powder X-ray diffractions of MOFs were obtained from a D/max-UltimaIII (Rigaku Corporation, Japan). X-ray photoelectron spectroscopy (XPS) measurements of MIL-101(Cr) and amine-MIL-101(Cr) were performed by a EscaLab 250Xi (Thermo Fisher Scientific, America). Nitrogen adsorption/desorption isotherms and pore size distributions of MOF scaffolds were collected at 77 K using V-Sorb2800P surface area and porosimetry analyzer (Gold APP Instruments Corporation, Beijing, China). High-pressure CO_2_ sorption measurements were carried out using an H-Sorb2600 high pressure and temperature gas sorption analyser (Gold APP Instruments Corporation, Beijing, China). The ^13^C spectrum of formic acid product was recorded on a 600 MHz Bruker AVANCE III (Bruker Corporation, Germany). A high-performance liquid chromatography (HPLC) 2030 system (Shimadzu, Kyoto, Japan) was applied to determine the concentrations of formate derivatives using a 5020-39001 WondaSil C_18_ column (15 × 4.6 cm i.d., 5 μm, GL Sciences) with UV detection at 280 nm.

### Synthesis of Amine-MIL-101(Cr)

MIL-101(Cr) was first synthesized by well dispersing 3.2 g of Cr(NO_3_)_3_·9H_2_O, 1.3 g of trimesic acid, and 687 μL of HCl in 40 mL water. The mixture was reacted at 220°C for 8 h. After the reaction was completed, the unreacted crystalline acid was removed and the product was collected by centrifugation at 12000 rpm for 6 min. The MIL-101(Cr) product was washed with ethanol for three times, and then activated by keeping in 40 mL of 95% ethanol at 80°C for 8 h. The final MIL-101(Cr) product was dried under vacuum at 160°C for 8 h before further use.

MIL-101(Cr) was then modified with different amines including hexamethylenediamine (HMD), cystamine, and branched polyethyleneimine. Dried MIL-101(Cr) with an amount of 0.2 g was first well-dispersed in anhydrous methanol, and the amine with a weight ratio of 1:1 or 1:2 was added to the solution. The mixture was reacted for 10 min. The collected amine-MIL-101(Cr) was washed with methanol for 3 times and then dried at 120°C for 6 h before further use.

### Synthesis of HKUST-1@amine-MIL-101(Cr)

For the preparation of HKUST-1@amine-MIL-101(Cr), Cu^2+^@amine-MIL-101(Cr) was first synthesized by well dispersing 30 mg amine-MIL-101(Cr) in 1 mL deionized water followed by the addition of 1 mL 50.1 mmol/L copper(II) acetate. After magnetic stirring at 1,200 rpm and 60°C for 1 h, the Cu^2+^@amine-MIL-101(Cr) product was thoroughly washed with water and collected by centrifugation. The Cu^2+^@amine-MIL-101(Cr) nanoparticles were then re-dispersed in 1 mL water, and 1 mL of 99.9 mmol/L 1,3,5-benzenetricarboxylic acid (H_3_BTC) was added to the solution. The mixture was reacted at 60°C for 1 h. After washing with methanol and water, the H_3_BTC@Cu^2+^@MIL-101(Cr) nanoparticles were re-dispersed in 1 mL water and mixed with 50.1 mmol/L copper(II) acetate and 99.9 mmol/L H_3_BTC. After reacting at 25°C for 2 h, the final H_3_BTC@Cu^2+^@MIL-101(Cr) product was washed thoroughly with water and collected by centrifugation.

### Immobilization of Enzymes in HKUST-1@amine-MIL-101(Cr)

For enzymatic catalysis of CO_2_ reduction with NADH regeneration, three enzymes including carbonic anhydrase (CA), formate dehydrogenase (FateDH), and glutamate dehydrogenase (GDH) were immobilized in HKUST-1@amine-MIL-101(Cr) with HKUST-1 in layered structure. CA was encapsulated in the inner HKUST-1 layer, and FateDH and GDH were immobilized in the outer HKUST-1 shell.

H_3_BTC@Cu^2+^@MIL-101 nanoparticles were first prepared following the above synthetic approach. The first HKUST-1 layer encapsulated with CA was then synthesized by reacting 1 mL aqueous solution containing 50.1 mmol/L copper(II) acetate, 99.9 mmol/L H_3_BTC, and 5 mg CA at 25°C for 2 h. After washing with water, the bioconjugates were mixed with 1 mL aqueous solution comprising 50.1 mmol/L copper(II) acetate, 99.9 mmol/L H_3_BTC, 3 mg FateDH, and 3 mg GDH. The mixture was reacted for another 2 h to generate the second HKUST-1 layer containing FateDH and GDH. The final product was washed thoroughly with water and collected by centrifugation.

### CO_2_ Storage

High pressure CO_2_ adsorption experiments were performed at 298.15 K and at pressure of 0–30 bar for 24 h using the H-Sorb2006 high pressure and temperature gas adsorption analyser. Before the adsorption of CO_2_, the MOFs (500 mg) were dried in a sample tube under vacuum at a temperature of 120°C overnight.

### Enzymatic Catalysis of CO_2_ to Formic Acid

For the enzymatic catalysis of stored CO_2_ to formic acid using immobilized enzymes, the HKUST-1@amine-MIL-101(Cr) nanocomposites were dried using freeze-drying and used for the adsorption of CO_2_ at 298.15 K and 5 bar for 24 h. A mixture solution containing 10 mM L-glutamate and 2 mg/mL NADH in 6 mL of 50 mM phosphate buffer saline solution was purged with nitrogen for 0.5 h to remove the dissolved air. And 30 mg of HKUST-1@amine-MIL-101(Cr)-based multienzymes with stored CO_2_ was quickly added to the above solution. The cascade reaction was performed in a sealed flask at 25°C for different times.

For the enzymatic catalysis of bubbled CO_2_ using immobilized enzymes, a mixture solution containing 10 mM L-glutamate and 2 mg/mL NADH in 6 mL of 50 mM phosphate buffer saline solution was purged with nitrogen for 0.5 h to remove the dissolved air, and then was bubbled with CO_2_ for 1 h. And 30 mg of HKUST-1@amine-MIL-101(Cr)-based multienzymes were quickly added to the above solution. The reaction was performed in a sealed flask at 25°C for 6 h.

For the enzymatic catalysis of bubbled CO_2_ using free enzymes, a mixture solution containing 10 mM L-glutamate and 2 mg/mL NADH in 6 mL of 50 mM phosphate buffer saline solution was purged with nitrogen for 0.5 h to remove the dissolved air, and then was bubbled with CO_2_ for 1 h. And 5 mg CA, 3 mg FateDH, and 3 mg GDH were quickly added to the above solution. The reaction was performed in a sealed flask at 25°C for 6 h.

After reaction, the supernatant was collected by centrifugation. The formic acid product was derivatized by mixing 200 μL of the sample, 100 μL of 100 mM Na_2_HPO_4_, and 400 μL of 20 mg/mL pentafluorobenzyl bromide in acetone, and reacted at 60°C for 1 h. The derivatized product was detected by HPLC.

## Results and Discussion

### Amine-Functionalized MIL-101(Cr) for the Storage of CO_2_

**Figure 1:**
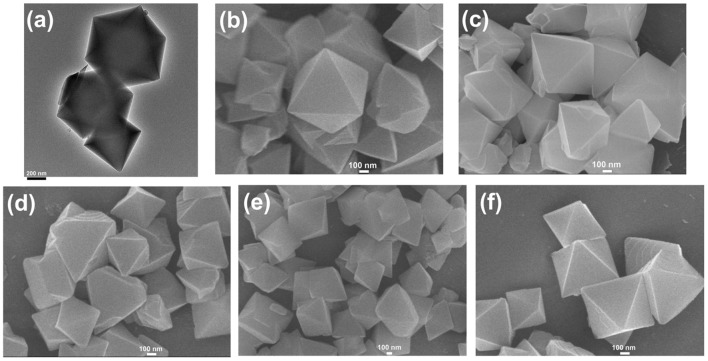
Transmission electron microscopy (TEM) image of MIL-101(Cr) **(a)**, and scanning electron microscopy (SEM) images of MIL-101(Cr) **(b)**, HMD-MIL-101(Cr) **(c)**, cystamine-MIL-101(Cr) **(d)**, PEI(50)-MIL-101(Cr) **(e)**, and PEI(100)-MIL-101(Cr) **(f)**.

**Figure 2:**
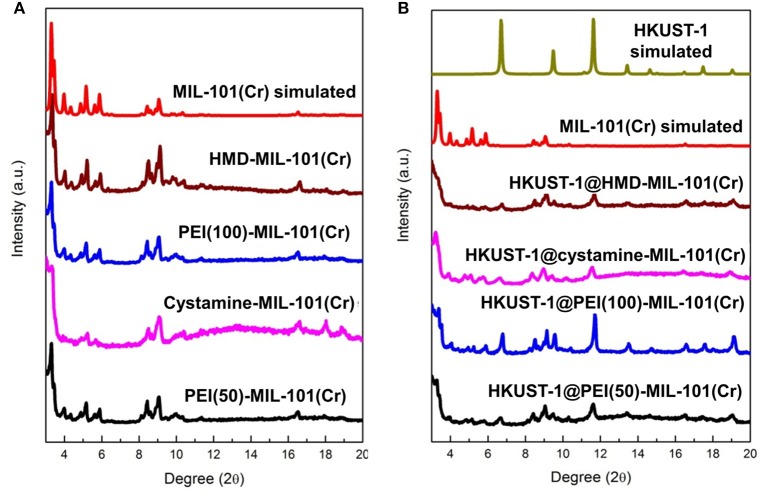
**(A)** X-ray diffraction patterns of MIL-101(Cr) simulated, HMD-MIL-101(Cr), PEI(100)-MIL-101(Cr), cystamine-MIL-101(Cr), and PEI(50)-MIL-101(Cr), and **(B)** X-ray diffraction patterns of HKUST-1 simulated, HKUST-1@HMD-MIL-101(Cr), HKUST-1@cystamine-MIL-101(Cr), HKUST-1@PEI(100)-MIL-101(Cr), and HKUST-1@ PEI(50)-MIL-101(Cr).

**Figure 3:**
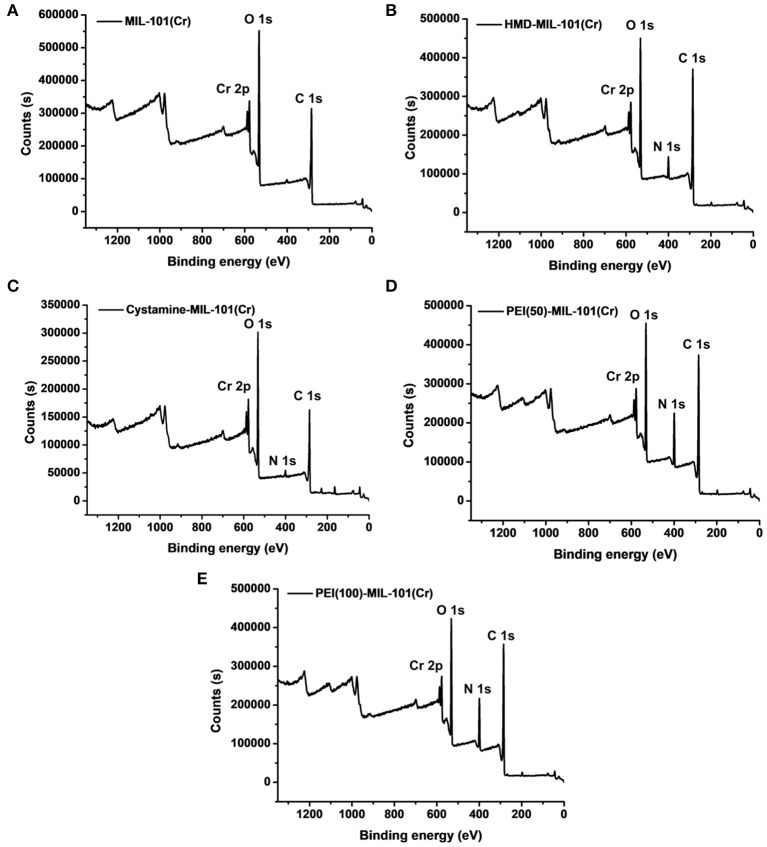
X-ray photoelectron spectroscopy measurements of MIL-101(Cr) **(A)**, HMD-MIL-101(Cr) **(B)**, cystamine-MIL-101(Cr) **(C)**, PEI(50)-MIL-101(Cr) **(D)**, and PEI(100)-MIL-101(Cr) **(E)**.

**Figure 4:**
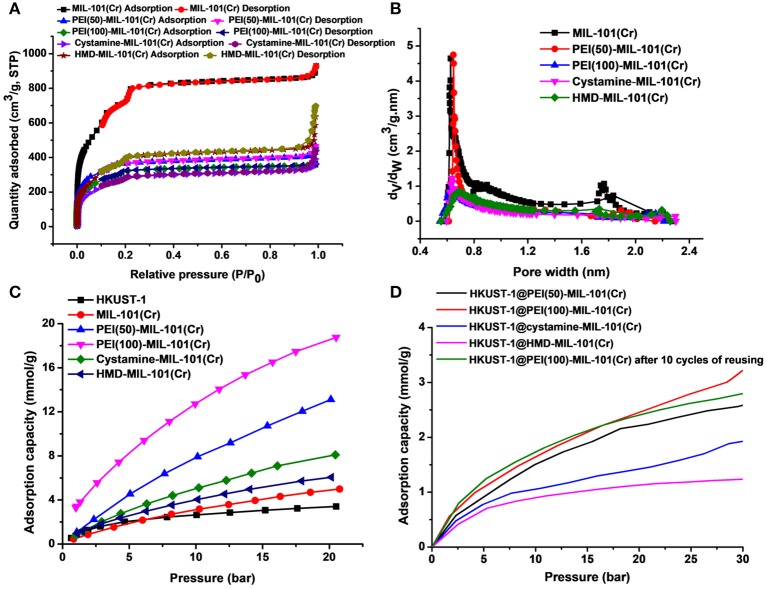
Nitrogen adsorption/desorption isotherms **(A)** and pore size distributions **(B)** of MIL-101(Cr) and its amine-functionalized counterparts. **(C)** CO_2_ adsorption capacities of HKUST-1, MIL-101(Cr), PEI(50)-MIL-101(Cr), PEI(100)-MIL-101(Cr), cystamine-MIL-101(Cr), and HMD-MIL-101(Cr). **(D)** CO_2_ adsorption capacities of HKUST-1@PEI(50)-MIL-101(Cr), HKUST-1@PEI(100)-MIL-101(Cr), HKUST-1@cystamine-MIL-101(Cr), and HKUST-1@HMD-MIL-101(Cr).

The transmission electron microscopy (TEM) and scanning electron microscopy (SEM) images in Figures 1a,b exhibited the octahedral morphology of MIL-101(Cr) nanocrystals with apparent corners and edges, which were in good agreement with literatures (Férey et al., 2005; Hwang et al., 2008). Amine-functionalized MIL-101(Cr) was obtained by the modification of MIL-101(Cr) with a series of amines including HMD, cystamine, and branched PEI with different loadings (50% and 100%) [here denoted as amine-MIL-101(Cr)]. We found that the original morphology of MIL-101(Cr) was preserved after loading of amines confirming that the amine functionalization step had little damage to the generic MOF (Figures 1c–f). The powder X-ray diffraction (PXRD) patterns of amine-MIL-101(Cr) were also essentially identical with its pristine counterpart indicating that the high crystallinity and purity of MOF were well-maintained (Figure 2A). However, the intensity of diffraction peaks below 7° decreased due to the filling of MOF pores by amines. The similar phenomenon has been observed by other researchers (Lin et al., 2013; Lin Y. et al., 2014). XPS in Figure 3A revealed 6.0 at% Cr, 59.6 at% C, and 34.4 at% O in MIL-101(Cr), which corresponded well with its molecular formula C_24_O_16_H_17_Cr_3_ (Férey et al., 2005). After functionalization with different amines, the appearances of 13.6, 7.25, 21.6, and 22.4 at% N elements were observed in HMD-MIL-101(Cr), cystamine-MIL-101(Cr), PEI(50)-MIL-101(Cr), and PEI(100)-MIL-101(Cr), respectively, confirming the successful postsynthetic modification (Figures 3B–E). We also measured the nitrogen adsorption/desorption isotherms at 77 K and pore size distributions of pristine and amine-MIL-101(Cr) as depicted in Figure 4. As expected, the postsynthetic modification of MIL-101(Cr) with different amines significantly reduced the specific surface areas and pore volumes as the amine functionalities occupied partial pore space of MOFs. While the surface area of MIL-101(Cr) was 2,477 m^2^/g, this value was decreased remarkably to 1,922, 1,011, 1,314, and 1,160 m^2^/g for HMD-MIL-101(Cr), cystamine-MIL-101(Cr), PEI(50)-MIL-101(Cr), and PEI(100)-MIL-101(Cr) (Figure 4A). The pore size distribution in Figure 4B revealed that MIL-101(Cr) had two types of micropores with a pore width of 0.63 and 1.77 nm, respectively. The pore volume was 1.44 cm^3^/g. Surface modification with amines significantly decreased the number of larger pores. As shown in Figure 4B, the pore volume of bigger pores at around 1.77 nm in amine-MIL-101(Cr) was significantly decreased compared with generic MIL-101(Cr). And the pore volume for HMD-MIL-101(Cr), cystamine-MIL-101(Cr), PEI(50)-MIL-101(Cr), and PEI(100)-MIL-101(Cr) was reduced to 1.08, 0.68, 0.72, and 0.56 cm^3^/g (Table 1).

**Table 1:** The BET surface area and pore volume of MIL-101(Cr) and amine-MIL-101(Cr).

**MOF**	**BET surface area (m^**2**^/g)**	**Pore volume (cm^**3**^/g)**
MIL-101(Cr)	2,477	1.44
HMD-MIL-101(Cr)	1,922	1.08
Cystamine-MIL-101(Cr)	1,011	0.68
PEI(50)-MIL-101(Cr)	1,314	0.72
PEI(100)-MIL-101(Cr)	1,160	0.56

Then we tested the gas adsorption performance of amine-MIL-101(Cr) for CO_2_. The CO_2_ adsorption isotherm at 298 K was illustrated in Figure 4C, and the results of CO_2_ sorption data at 5 bar and 298 K for four amine-MIL-101(Cr) were shown in Table 2. Apparently, amine-MIL-101(Cr) showed much higher adsorption capacity for CO_2_ compared with parent MIL-101(Cr). At 5 bar and 273 K, the CO_2_ adsorption capacity of PEI(100)-MIL-101(Cr) reached 8.25 mmol/g, which was 4.4-fold higher than that observed in MIL-101(Cr). Similarly, the CO_2_ adsorption capacities of HMD-MIL-101(Cr), cystamine-MIL-101(Cr), and PEI(50)-MIL-101(Cr) was 2.57, 3.11, and 4.48 mmol/g, respectively, which was 1.4~2.4 fold higher than that of unmodified MIL-101(Cr). The enhancement of CO_2_ storage capacity may be ascribed to the introduction of amine functionalities in the MOF pore environment, which donates electrons and improves the affinity of MOF materials toward CO_2_ molecules via dipole–quadrupole interactions (Zheng et al., 2011). Clearly, high loading of branched PEI provided more amine functionalities in MIL-101(Cr) according to the XPS results in Figure 3, which facilitated the enhancement of CO_2_ capture capacity. As a result, PEI(100)-MIL-101(Cr) exhibited the highest adsorption capacity for CO_2_. For comparison, we also tested the adsorption capacity of HKUST-1 for CO_2_, which was only 2.17 mmol/g at 5 bar and 298 K.

**Table 2:** The adsorption capacity of amine-MIL-101(Cr) for CO_2_ at 5 bar and 298 K.

**MOF**	**Adsorption capacity for CO_2_ (mmol/g)**
MIL-101(Cr)	1.88
HMD-MIL-101(Cr)	2.57
Cystamine-MIL-101(Cr)	3.11
PEI(50)-MIL-101(Cr)	4.48
PEI(100)-MIL-101(Cr)	8.25
HKUST-1	2.17
HKUST-1@HMD-MIL-101(Cr)	0.65
HKUST-1@Cystamine-MIL-101(Cr)	0.80
HKUST-1@PEI(50)-MIL-101(Cr)	0.94
HKUST-1@PEI(100)-MIL-101(Cr)	1.14

### Construction of Multienzymatic Cascade System

Three enzymes including CA, FateDH, and GDH were immobilized in HKUST-1 using a layer-by-layer self-assembly approach. HKUST-1 was selected as the solid support for the immobilization of enzymes because of its good solvent tolerance and mild preparative conditions. To fully utilize the stored CO_2_ as substrate, the multienzyme system was constructed on the surface of amine-MIL-101(Cr). The enzymes were co-immobilized in HKUST-1 with layered structure to achieve the channeling of substrate. As illustrated in Scheme 1, using amine-MIL-101(Cr) as the core, the first HKUST-1 layer encapsulated with CA was fabricated followed by the second HKUST-1 layer containing FateDH and GDH. In this case, the CO_2_ substrate released from amine-MIL-101(Cr) first got access to CA and were hydrated to bicarbonate ions. The ${\text{HCO}}_{3}^{-}$ intermediate then migrated directly to the FateDH enzyme and was converted to formic acid. GDH in the outer MOF shell was used to achieve the *in situ* regeneration of NADH co-factor for the continuous production of formic acid. The enzyme immobilization capacity was 267.4 mg/g for CA, and 669.6 mg/g for FateDH and GDH. It is worthy of note that the size of micropores of amine-MIL-101(Cr) does not match the large dimensions of enzymes. As a result, the immobilization of enzymes will not affect the CO_2_ adsorption capacities of amine-MIL-101(Cr).

**Figure 5:**
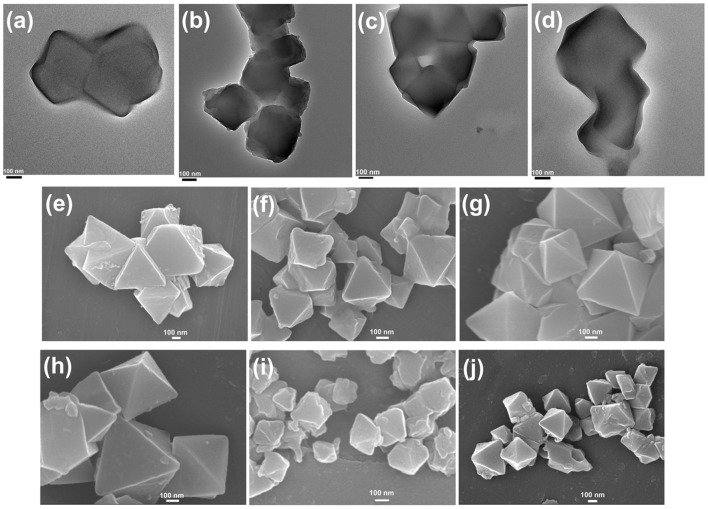
Transmission electron microscopy images of HKUST-1@HMD-MIL-101(Cr) **(a)**, HKUST-1@cystamine-MIL-101(Cr) **(b)**, HKUST-1@PEI(50)-MIL-101(Cr) **(c)**, and HKUST-1@PEI(100)-MIL-101(Cr) **(d)**. Scanning electron microscopy (SEM) images of HKUST-1@HMD-MIL-101(Cr) **(e)**, HKUST-1@cystamine-MIL-101(Cr) **(f)**, HKUST-1@PEI(50)-MIL-101(Cr) **(g)**, HKUST-1@PEI(100)-MIL-101(Cr) **(h)**, immobilized enzymes in HKUST-1@PEI(100)-MIL-101(Cr) (i), and immobilized enzymes in HKUST-1@PEI(100)-MIL-101(Cr) after repeated use for 10 cycles **(j)**.

As shown in Supplementary Figure 1, the formation of HKUST-1 on the surface of amine-MIL-101(Cr) turned the MOF aqueous solution from green to blue-green. Energy-dispersive X-ray spectroscopy (EDS) analysis revealed the appearance of 9.98, 2.53, 10.15, and 18.62 at% Cu in HKUST-1@HMD-MIL-101(Cr), HKUST-1@cystamine-MIL-101(Cr), HKUST-1@PEI(50)-MIL-101(Cr), and HKUST-1@PEI(100)-MIL-101(Cr), respectively, implying the formation of HKUST-1 layer. The XRD patterns of HKUST-1@amine-MIL-101(Cr) illustrated in Figure 2B revealed new peaks typical of HKUST-1 nanocrystals. Further characterizations with TEM (Figures 5a–d) and SEM (Figures 5e–h) also confirmed the successful generation of HKUST-1@amine-MIL-101(Cr) nanocomposites.

We next evaluated the gas adsorption capacity of HKUST-1@amine-MIL-101(Cr) for CO_2_. As shown in Figure 4D, the HKUST-1@amine-MIL-101(Cr) had much lower storage capacity for CO_2_ presumably as a result of the partial filling of the micropores. But this storage capacity for CO_2_ is still superior than using bubbled CO_2_ as its solubility in water is only of 33 mM (Zhang Z. et al., 2018).

### Conversion of CO_2_ to Formic Acid

**Figure 6:**
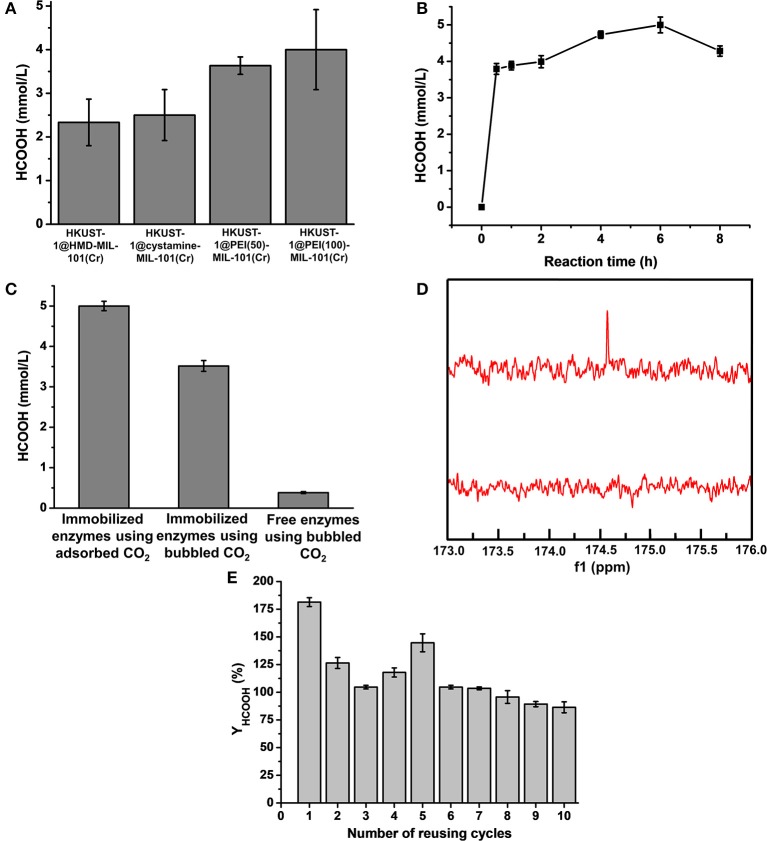
**(A)** Production amount of HCOOH catalyzed by HKUST-1@HMD-MIL-101(Cr), HKUST-1@cystamine-MIL-101(Cr), HKUST-1@PEI(50)-MIL-101(Cr), and HKUST-1@PEI(100)-MIL-101(Cr) immobilized enzyme systems. **(B)** Formic acid production at different reaction times. **(C)** Production amount of HCOOH catalyzed by HKUST-1@PEI(100)-MIL-101(Cr) immobilized enzymes using adsorbed CO_2_ as substrate, HKUST-1@PEI(100)-MIL-101(Cr) immobilized enzymes using bubbled CO_2_ as substrate, and free enzymes using bubbled CO_2_ as substrate. **(D)** ^13^C NMR spectrum of formic acid produced from HKUST-1@PEI(100)-MIL-101(Cr) immobilized enzymes using adsorbed CO_2_ as substrate. **(E)** Reusability of HKUST-1@PEI(100)-MIL-101(Cr) immobilized enzymes with respect to the number of reaction cycles in which the adsorbed CO_2_ was used as substrate.

The newly constructed HKUST-1@amine-MIL-101(Cr)-based multienzymes containing CA, FateDH, and GDH were employed to reduce CO_2_ to formic acid using the stored CO_2_ as the starting substrate accompanied by NADH regeneration. The HCOOH synthesis reaction was carried out in batch mode containing 30 mg of MOF-based multienzymes and 2.2 mmol/L NADH in 6 mL reaction system. The preliminary reaction time was 2 h. The formic acid produced from the four multienzyme systems were calculated and compared in Figure 6A and Supplementary Table 1. Clearly, larger CO_2_ adsorption capacity of MOFs corresponded to higher HCOOH production yield. The HKUST-1@PEI(100)-MIL-101(Cr) multienzyme system exhibited the highest HCOOH production amount of 4.0 ± 0.92 mmol/L due to its largest adsorption capacity for CO_2_, which was 24 ± 5.5 μmol. This is equal to 71.1% conversion yield taking into consideration that 33.75 μmol of CO_2_ was stored in HKUST-1@PEI(100)-MIL-101(Cr) at 5 bar and 298 K.

To further increase the conversion yield of CO_2_, we also optimized the reaction time. The production amount of HCOOH from the adsorbed CO_2_ catalysed in HKUST-1@PEI(100)-MIL-101(Cr) multienzyme system was depicted as a function of reaction time. As shown in Figure 6B and Supplementary Table 2, the highest HCOOH amount of 5.0 ± 0.22 mmol/L was obtained at a reaction time of 6 h which represented a conversion yield of 88.9%. Obviously, the stored CO_2_ was not completely transformed to formic acid. One of the possible reason is the partial release of CO_2_ because the whole enzymatic catalysis process is performed at 1 bar. We also observed that the produced HCOOH amount decreased with the elongation of reaction time. This can be partly explained by the fact that the reaction rate of CO_2_ to HCOOH catalysed by FateDH is much slower than its reverse reaction (HCOOH to CO_2_) (Rusching et al., 1976; Zhang Z. et al., 2018). As we know, the production of 1 mol formic acid consumes 1 mol NADH. When the regeneration of NADH catalysed by GDH is not as effective as its consumption, the deficiency of NADH may cause the transformation of formic acid to CO_2_.

For comparison, we also performed the enzymatic reactions catalyzed by immobilized enzymes and free enzymes using bubbled CO_2_ as the substrate. As shown in Figure 6C, the production amount of HCOOH catalysed by free enzymes using bubbled CO_2_ as substrate was only 0.38 ± 0.03 mmol/L. By using the immobilized enzymes to catalyse the bubbled CO_2_, the produced HCOOH increased to 3.52 ± 0.13 mmol/L. The conversion using bubbled CO_2_ as substrate was also calculated based on the CO_2_ solubility of 33 mM in water (Zhang Z. et al., 2018), which was only 10.67% for immobilized enzymes and 1.15% for free enzymes, far <100%. Clearly, the produced HCOOH catalysed by the immobilized multienzyme system using stored CO_2_ as substrate was more than 13.1-times higher than that of the corresponding free enzyme systems. These results clearly demonstrated the superiority of our new strategy. The immobilization of enzymes in HKUST-1 layered structure is kinetically advantageous over free enzymes. The adsorbed CO_2_ was gradually released from amine-MIL-101(Cr) and was directly converted to bicarbonate ions by CA which was encapsulated in the inner layer. The intermediate bicarbonate ions were then *in situ* consumed by FateDH immobilized on the outer MOF layer without diffusion through long distance. The porous structure of MOF allowed efficient diffusions of substrate and products. This synthetic route facilitated the channeling of substrate and eventually enabled higher rate of the cascade reaction. Moreover, the use of adsorbed CO_2_ as substrate provided CA and FateDH with a high CO_2_ concentration stored in a slow-releasing MOF system as required by CA and FateDH, which allowed much more production of formic acid.

To further demonstrate that the formic acid was produced from catalysing the CO_2_ adsorbed in MOFs instead of free CO_2_ in the air. ^13^CO_2_ was stored in MOFs and used as substrate catalysed by HKUST-1@PEI(100)-MIL-101(Cr)-based multienzymes. The final product was analyzed by ^13^C NMR. Figure 6D displayed the prominent peak of ^13^C at 174.6 ppm which belonged to H^13^COOH. The surface morphology of HKUST-1@PEI(100)-MIL-101(Cr) immobilized enzymes was also characterized by SEM. As shown in Figure 5i, the immobilization of enzymes did not change the shape and morphology of MOF scaffolds.

### NADH Regeneration With Glutamate Dehydrogenase (GDH)

NADH is the co-factor functioning as a terminal electron donor and hydrogen donor in the cascade enzymatic reaction. The production of 1 mol formic acid from CO_2_ consumes 1 mol costly NADH generating NAD^+^. As the presence of NAD^+^ suppresses the reduction of CO_2_ to formic acid and accelerates its reverse oxidation reaction, the efficient regeneration of NADH is highly desirable. Enzymes such as glucose dehydrogenase (Obón et al., 1998; Marpani et al., 2017; Zhang Z. et al., 2018), xylose dehydrogenase (Marpani et al., 2017) and GDH (Ji et al., 2015) have been successfully used for the regeneration of NADH. In our work, GDH was adopted to attain the continuous conversion of NAD^+^ to NADH.

We investigated the effects of NADH concentration on the overall reaction efficiency by varying the added NADH amount in the reaction solution at a final concentration between 0.5 and 2.8 mM while keeping the immobilized enzymes amount constant. The NADH-based HCOOH yield (*Y*_HCOOH_) was calculated according to the following equation.

(1) $$\begin{array}{r} Y_{HCOOH}\left(\%\right)=\frac{C_{HCOOH}}{C_{NADH,\;initial}}\times 100 \end{array}$$

where *C*_HCOOH_ is the HCOOH concentration (mM) at a reaction time of 6 h, and *C*_*NADH, initial*_ is the initial NADH concentration (mM).

As shown in Table 3, the production of HCOOH raised up to 5.04 mM when the NADH concentration increased from 0.5 to 2.8 mM, while *Y*_HCOOH_ decreased from 353.88 to 179.82%. This trend was similar to the work reported by Zhang et al. in which the sequential co-immobilization of five enzymes in hollow nanofiber was achieved and used for the synthesis of methanol from CO_2_ (Ji et al., 2015). As reported by Pinelo et al. (Zhang Z. et al., 2018), the reaction rate for reducing NAD^+^ to NADH is much higher than its reverse oxidation reaction catalysed by FateDH. The same finding was also observed in our work. NADH was efficiently regenerated by GDH encapsulated in the outer MOF shell. The catalytic performance of our newly designed HKUST-1@PEI(100)-MIL-101(Cr) immobilized systems compares well with the values obtained from other immobilized enzymes published by other groups shown in Table 4.

**Table 3:** HCOOH production at different NADH concentration.

**NADH (mM)**	**HCOOH (mM)**	***Y*_**HCOOH**_ (%)**
0.5	1.77	353.9
1	2.83	283.3
2	3.21	160.6
2.8	5.04	179.8

**Table 4:** Comparison of the NADH-based methanol or HCOOH yield produced using HKUST-1@PEI(100)-MIL-101(Cr) immobilized enzymes and other immobilized systems reported in published literatures.

**Immobilization matrix**	**Enzyme^a^**	**Initial concentration of NADH**	***Y*_**(Product, t)**_ (%)**	**References**
Polystyrene particles	FateDH, FaldDH, ADH, GDH	50 μM	52.6^b^	El-Zahab et al., 2008
GelCSi hybrid microcapsules	FateDH, FaldDH, YADH	50 mM	71.6^b^	Wang D. et al., 2014
Porous silica sol-gel	FateDH, FaldDH, ADH	50 μM	91.2^b^	Obert and Dave, 1999
Alginate-silica hybrid gel	FateDH, FaldDH, ADH	940 μM	98.1^b^	Xu et al., 2006
Hollow nanofiber membrane	FateDH, FaldDH, ADH	1 mM	103.2^b^	Ji et al., 2015
ZIF-8	FateDH,GDH, FaldDH, ADH	10 mM	40.2^b^	Zhu et al., 2019
Titania nanoparticle	FateDH, FaldDH,	50 mM	92.7^b^	Shi et al., 2012
Millimeter-scale gel bead	FateDH, FaldDH, ADH	0.1 mM	22.5^b^	Jiang et al., 2009
HKUST-1@PEI(100)-MIL-101(Cr)	CA, FateDH, GDH	0.1 mM	353.9^c^	This work

a *Formate dehydrogenase (FateDH), formaldehyde dehydrogenase (FaldDH), alcohol dehydrogenase (ADH), glutamate dehydrogenase (GDH), and yeast alcohol dehydrogenase (YADH)*.

b *The final product was methanol*.

c *The final product was formic acid*.

### Operational Stability and Reusability

The operational stability and reusability of enzymes immobilized in HKUST-1@PEI(100)-MIL-101(Cr) were evaluated by testing the HCOOH production amount after repeated catalysis of adsorbed CO_2_ for 10 cycles. After one batch of reaction for 6 h, the MOF scaffold containing enzymes were dried using freeze drying and used for the adsorption of CO_2_ at 5 bar and 298 K before the next batch of catalysis. As shown in Figure 6E, the NADH-based HCOOH yield (*Y*_HCOOH_) was still 86% even after 10 cycles of reusing. A cumulative HCOOH yield of 1077.7% was obtained from the 10 reusing cycles of this reaction system indicating the good operational stability and reusability of the immobilized enzymes.

We also tested the chemical tolerance of the MOF scaffold. The HKUST-1@PEI(100)-MIL-101(Cr) nanocomposite obtained from 10 cycles of reusing was subjected to SEM measurement. As shown in Figure 5j, there was no change in the morphology of MOF support indicating its high chemical stability. The gas storage capacity of HKUST-1@PEI(100)-MIL-101(Cr) nanocomposite after 10 cycles of reusing was also evaluated. As illustrated in Figure 4D, the repeated reaction did not lead to any decrease in the CO_2_ uptake capacity thus confirming the reusability of the MOFs as adsorbent for the storage of CO_2_.

## Conclusions

We have developed a new MOF scaffold that functions as adsorbent for the storage of CO_2_ as well as solid support for the sequential co-immobilization of multienzymes via a layer-by-layer self-assembly approach. This new strategy used the adsorbed CO_2_ as substrate, facilitated the channeling of substrate, and eventually enabled high catalytic efficiency with a continuous regeneration of NADH co-factor. Improved operational stability and reusability were also observed in immobilized enzymes implying the great potential of our new strategy for the biotransformation of CO_2_ used in industrial applications.

## Data Availability Statement

All datasets generated for this study are included in the article/Supplementary Material.

## Author Contributions

YLi and LW carried out the experiments. YLv and TT conceived and designed the experiments. YLv and YLi analyzed the data and wrote the manuscript.

### Conflict of Interest

The authors declare that the research was conducted in the absence of any commercial or financial relationships that could be construed as a potential conflict of interest.
