# Supplementary material for: Sequential Co-immobilization of Enzymes in Metal-Organic Frameworks for Efficient Biocatalytic Conversion of Adsorbed CO2 to Formate
Source: Front Bioeng Biotechnol. 2019 Dec 6;7:394. doi: 10.3389/fbioe.2019.00394 (PMC6908815; doi:10.3389/fbioe.2019.00394)
Supplement: Supplementary file 1 [file Table_1.DOCX]

Sequential co-immobilization of enzymes in metal-organic frameworks for efficient biocatalytic conversion of adsorbed CO_2_ to formate

Yan Li, Liyin Wen, Tianwei Tan, Yongqin Lv*

## Supplementary Figures


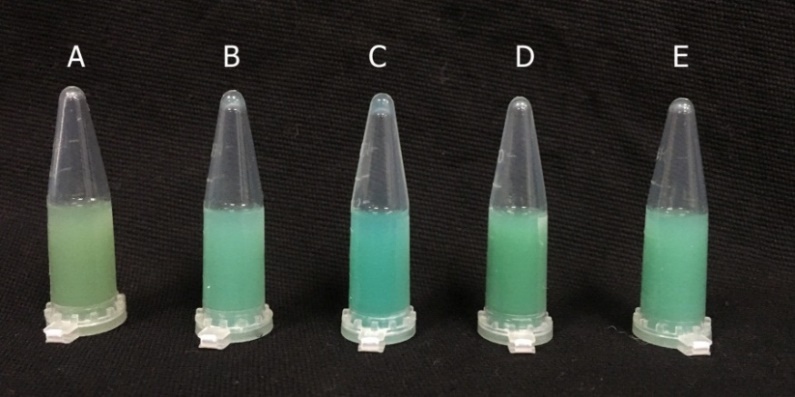


**Supplementary Figure 1.** Photographs of aqueous solutions of MIL-101(Cr) (A), HKUST-1@PEI(50)-MIL-101(Cr) (B), HKUST-1@PEI(100)-MIL-101(Cr) (C), HKUST-1@cystamine-MIL-101(Cr) (D), and HKUST-1@HMD-MIL-101(Cr) (E).

## Supplementary Tables

**Supplementary Table 1.** Production amount of HCOOH catalysed by HKUST-1@HMD-MIL-101(Cr), HKUST-1@cystamine-MIL-101(Cr), HKUST-1@PEI(50)-MIL-101(Cr), and HKUST-1@PEI(100)-MIL-101(Cr) immobilized enzyme systems.

| Immobilized enzymes | Enzyme dosage (mg) | | | HCOOH (mmol/L) |
| --- | --- | --- | --- | --- |
|  | CA | FateDH | GDH |  |
| HKUST-1@HMD-MIL-101(Cr) | 5 | 3 | 3 | 2.33±0.53 |
| HKUST-1@cystamine-MIL-101(Cr) | 5 | 3 | 3 | 2.50±0.58 |
| HKUST-1@PEI(50)-MIL-101(Cr) | 5 | 3 | 3 | 3.63±0.20 |
| HKUST-1@PEI(100)-MIL-101(Cr) | 5 | 3 | 3 | 4.00±0.92 |

**Supplementary Table 2.** Production amount of HCOOH catalysed by HKUST-1@PEI(100)-MIL-101(Cr) immobilized enzymes using adsorbed CO_2_ as substrate, HKUST-1@PEI(100)-MIL-101(Cr) immobilized enzymes using bubbled CO_2_ as substrate, and free enzymes using bubbled CO_2_ as substrate.

| Immobilized enzymes | Substrate | Enzyme dosage (mg) | | | HCOOH (mmol/L) |
| --- | --- | --- | --- | --- | --- |
|  |  | CA | FateDH | GDH |  |
| HKUST-1@PEI(100)-MIL-101(Cr) | Adsorbed CO_2_ | 5 | 3 | 3 | 5.00±0.12 |
| HKUST-1@PEI(100)-MIL-101(Cr) | Bubbled CO_2_ | 5 | 3 | 3 | 3.52±0.13 |
| Free enzymes | Bubbled CO_2_ | 5 | 3 | 3 | 0.38±0.03 |
